# Prolonged Response to Afatinib and Crizotinib in a Rare Case of *EGFR*-, *HER2*-, *MET*- and *ROS1*-Alterated Lung Adenocarcinoma

**DOI:** 10.3390/ijms25115698

**Published:** 2024-05-23

**Authors:** Eva Plomer, Martin Früh, Arno Lauber, Izadora Demmer, Wolfram Jochum, Kira-Lee Koster

**Affiliations:** 1 Department of Medical Oncology and Haematology, Cantonal Hospital St. Gallen, Rorschacher Strasse 95, 9007 St. Gallen, Switzerland; eva.plomer@kssg.ch (E.P.); martin.frueh@kssg.ch (M.F.); 2 Faculty of Medicine, University of Bern, Murtenstrasse 11, 3008 Bern, Switzerland; 3 Department of Radiology, Cantonal Hospital St. Gallen, Rorschacher Strasse 95, 9007 St. Gallen, Switzerland; arno.lauber@kssg.ch; 4 Institute of Pathology, Cantonal Hospital St. Gallen, Rorschacher Strasse 95, 9007 St. Gallen, Switzerland; izadora.demmerbuchs@kssg.ch (I.D.); wolfram.jochum@kssg.ch (W.J.)

**Keywords:** NSCLC, *EGFR*-mutated NSCLC, targeted therapies, drug combinations, molecular pathology

## Abstract

We present the case of a 70-year-old never-smoking female patient with *epidermal growth factor receptor* (*EGFR*) p.L858R-mutated metastatic non-small cell lung cancer (NSCLC). After three months of first-line treatment with erlotinib, progression occurred and platinum/pemetrexed was initiated, followed by a response for more than two years. After the progression, the molecular testing of a vertebral metastasis revealed a *ROS proto-oncogene 1* (*ROS1*) translocation and a *human epidermal growth factor receptor 2* (*HER*2) p.S310F mutation, in addition to the known *EGFR* p.L858R mutation. Crizotinib then led to a durable response of 17 months. The molecular retesting of the tumour cells obtained from the recurrent pleural effusion revealed the absence of the *ROS1* translocation, whereas the *EGFR* and *HER2* mutations were still present. Afatinib was added to the crizotinib, and the combination treatment resulted in another durable response of more than two years. The patient died more than 7 years after the initial diagnosis of metastatic NSCLC. This case demonstrates that the repeated molecular testing of metastatic NSCLC may identify new druggable genomic alterations that can impact the patient management and improve the patient outcome.

## 1. Introduction

The guidelines recommend repeated genomic tumour testing in metastatic non-small cell lung cancer (NSCLC) to guide the treatment decisions during the course of the disease [1,2]. The detection of targetable driver mutations ideally results in the treatment with targeted therapies. There is limited knowledge regarding the impact of the co-alterations that may alter the response to targeted therapies. Therefore, careful decision-making is crucial in this setting. 

## 2. Case Presentation

A 70-year-old never-smoking female presented with a cough and dyspnoea during exercise in October 2015. Computed tomography (CT) and 18F-fluorodeoxyglucose-positron emission tomography (FDG-PET) showed a spiculated nodule in the lingula of the lung, additional bilateral pulmonary nodules and enlarged locoregional lymph nodes. Primary adenocarcinoma of the lung was diagnosed by bronchoalveolar lavage (BAL) and biopsy of a bronchial mass (TNM staging (7th edition): cT4, cN3, cM1a, stage IV). The next-generation sequencing (NGS; Ion AmpliSeq Colon and Lung Cancer Research Panel v2) of the tumour DNA revealed a classical *epidermal growth factor receptor* (*EGFR*) mutation in exon 21 (p.L858R; *c.2573T > G*). Additionally, a *MET* amplification (mean gene copy number 9; MET/CEN7-ratio 2.4) was detected by fluorescence in situ hybridization (FISH). 

**Figure 1:**
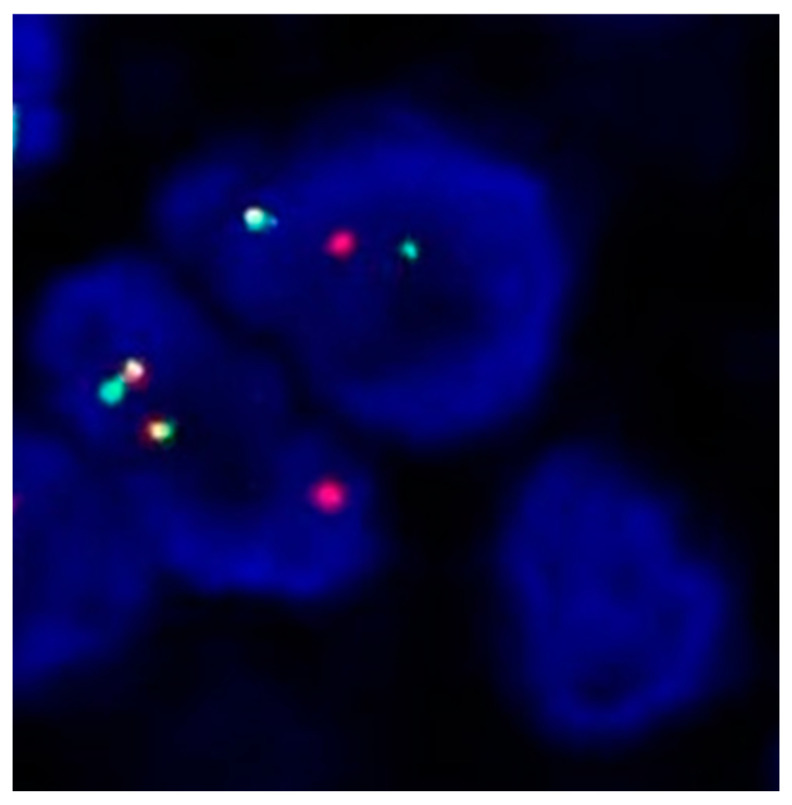
FISH analysis of NSCLC cells using *ROS1* dual colour break-apart probe. Nuclei show a signal pattern characterized by separate red and green signals indicative of *ROS1* translocation.

The patient was started on first-line erlotinib (150 mg daily) in November 2015, which led to a stable disease. In February 2016, the patient complained about increasing cough and dyspnoea due to pulmonary progression. The molecular testing of the carcinoma cells obtained by BAL showed no *EGFR* T790M mutation, and she was started on carboplatin and pemetrexed followed by pemetrexed maintenance, resulting in a partial remission for 32 months. In November 2018, the patient underwent spinal surgery for a pathological fracture of the 12th thoracic vertebra. Molecular testing (Oncomine Focus Assay) was performed on the epidural tumour tissue that was removed at the time of surgery. In addition to the initial *EGFR* p.L858R mutation, a *human epidermal growth factor receptor* 2 (*HER2)* mutation (p.S310F, *c.929C > T*) was discovered. Break-apart *ROS proto-oncogene 1 (ROS1)* FISH was positive for *ROS1* translocation (split signals in 39% of tumour cell nuclei, Figure 1). Furthermore, FISH revealed persistence of the *MET* amplification (mean gene copy number 4.6; MET/CEN7 ratio 2.2).

**Figure 2:**
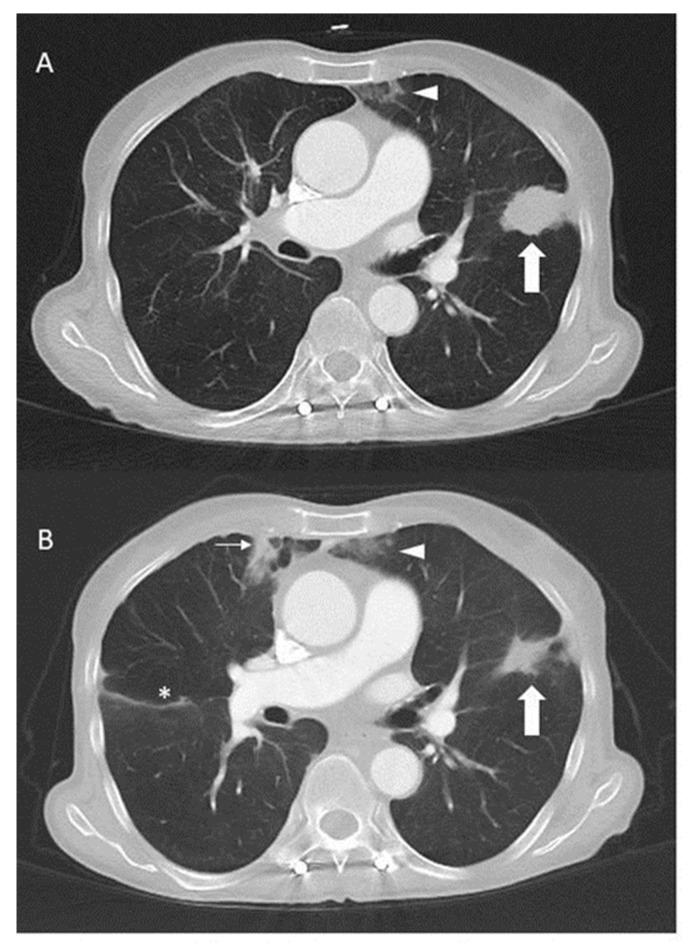
Computed tomography of the thorax with contrast before (**A**) and three months after the addition (**B**) of crizotinib to afatinib in October 2020. The mass (thick arrow) in the lingula responded to therapy. Incidental findings include subpleural consolidations (thin arrow) and dystelectasis (arrowheads) and slight interlobar effusion (*) on the right side.

Third-line crizotinib monotherapy (250 mg twice per day) was started in February 2019, resulting in a partial remission lasting 17 months until July 2020, when the disease again progressed in the lungs and pleura. The molecular testing (Oncomine Focus Assay) of the tumour cells from the pleural effusion showed persistent *EGFR* p.L858R and *HER2* p.S310F mutations, but no *ROS1* fusion transcripts. In addition, the *ROS1* FISH test was also negative. Due to the stable lesions interpreted as a response to the treatment outside of the thorax, crizotinib was continued and the pan-HER-inhibitor afatinib 30 mg daily was added to target EGFR and HER2 in October 2020. Crizotinib subsequently had to be reduced to 200 mg every other day due to oedema and afatinib to 20 mg at 2 out of 3 days due to skin toxicity. This time, a partial response with a clinical benefit could be observed for two years (Figure 2). 

**Figure 3:**
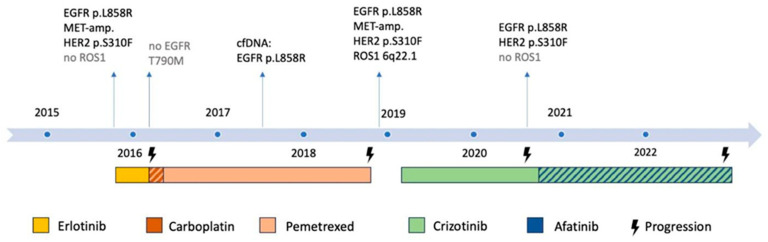
Overview of the molecular testing results and treatments during the course of disease (including important results from retrospective testing). The hatchings indicate the treatment with a drug combination.

Unfortunately, the patient deteriorated in November 2022 due to progressive disease and confusion as a result of likely paraneoplastic multiple infarct dementia and died in December 2022, more than seven years after the initial diagnosis of metastatic NSCLC. The molecular findings in the course of the disease are summarized in Figure 3. 

## 3. Discussion

In this case report, we describe the history of a never-smoking female patient with metastatic adenocarcinoma of the lung with an *EGFR* p.L858R mutation, a *HER2* p.S310F mutation and a *MET* amplification developing a *ROS1* translocation during the course of her disease. Our case illustrates the importance of repeated molecular testing upon disease progression in NSCLC patients treated with targeted therapy, which is also recommended in the international guidelines [1,2].

*EGFR* mutations occur in 10–20% of the lung adenocarcinomas in Western populations [3,4,5]. In our patient, the duration of disease control was rather short with erlotinib, a selective EGFR tyrosine kinase inhibitor (TKI). Although the outcome of the patients with the *EGFR* p.L858R mutation is inferior compared with *EGFR* exon 19 deletions, first-line therapy with erlotinib typically results in a progression-free survival in the range of 12 months [6,7,8]. One reason for the short duration of the response to erlotinib may have been the de novo *MET* amplification at the first diagnosis. *MET* amplifications are reported in approximately 5% of the patients with acquired resistance to first-generation EGFR TKIs [9,10,11] but are also rarely encountered as co-alterations in EGFR TKI-naïve patients [12,13]. The *HER2* p.S310F mutation may also have contributed to the reduced response to erlotinib in our patient [14]. This mutation had already been present at the time of diagnosis, as demonstrated by the post hoc NGS analysis using a larger gene panel. 

After the progression on EGFR TKI, the patient had a prolonged response to pemetrexed-based chemotherapy for more than two years. Surprisingly, at the time of the progression on pemetrexed, a *ROS1* translocation was found by FISH (39% of the tumour cells with a split signal) upon molecular NSCLC retesting. However, *ROS1* fusion transcripts could not be detected in a post hoc analysis of the tumour RNA using the FusionPlex Expanded Sarcoma Assay. The *ROS1* translocation had not been present upon the initial diagnosis, which has been confirmed by a recently performed post hoc test using the tumour cells obtained at the time of the first diagnosis. More favourable outcomes with pemetrexed have been observed in *ROS1*-altered NSCLC compared to *ALK-* or *EGFR*-positive disease [15]. Co-alterations of the *ROS1* and *EGFR* genes are exquisitely rare [16,17,18,19]. 

Based on the positive *ROS1* FISH result, a systemic therapy with crizotinib, a TKI inhibiting ROS1, MET and ALK, was started [20,21,22,23]. Crizotinib has been investigated in the PROFILE 1001 trial and shown to be effective in *ROS1*-altered NSCLC with durable responses (median duration of response 24.7 months; 95% confidence interval (CI) 15.2–45.3) [21]. Our patient was able to clinically benefit for 2 years as well, suggesting that, indeed, ROS1, rather than MET, was likely the driver of the tumour progression. The patient developed new pleural effusions under the systemic treatment with crizotinib after approximately 15 months. Interestingly, the *ROS1* translocation disappeared, whereas the known *EGFR* and *HER2* mutations were still present. The addition of afatinib, a pan-HER-inhibitor, to crizotinib with the intention to target the *HER2* and *EGFR* mutation led to another prolonged response of over 2 years [24]. Afatinib shows some activity not only against classical *EGFR* but also *HER2*-mutated tumours [25,26]. In addition, afatinib has been used successfully in a patient with metastatic lung adenocarcinoma harbouring an *EGFR* p.L858R mutation co-occurring with the same *HER2* p.S310F mutation [27]. The preferred approach of the concurrent inhibition of all the detected alterations with a combination therapy rather than a single-agent therapy against the postulated resistance mutation has been recently shown in the INSIGHT 2 study [28,29]. In this study, patients with *EGFR*-mutated NSCLC and a *MET* amplification as the resistance mechanism showed a much higher response rate (ORR) with the combination of osimertinib and tepotinib compared to tepotinib alone (54.5% [95% CI 32.2–75.6] vs. 8.3% [95% CI 0.2–38.5]) [28]. 

According to this observation, nowadays, one would have preferably treated this patient with the combination of afatinib and crizotinib from the beginning of the detection of the resistance mutations.

Our case underlines the importance of repeated molecular testing in NSCLC treated with targeted therapies in order to individualise the systemic treatment in the course of the disease [1,2]. Due to tumour heterogeneity, mixed responses and oligoprogression on the EGFR TKIs may occur when subclonal tumour cells harbouring different *EGFR* or other gene alterations co-exist with tumour cells still harbouring sensitive *EGFR* mutations [30,31,32]. 

With broader and deeper molecular testing, the presence of co-mutations and the emergence of novel resistance mutations during TKI therapy are becoming increasingly challenging in clinical practice. Nowadays, the first-line treatment of patients with lung cancer harbouring a classical *EGFR* mutation (exon 19 deletion or L858R mutation in exon 21) is usually osimertinib, a third-generation irreversible EGFR TKI, with or without chemotherapy as the first-line therapy [33,34]. The emergence of *ROS1* rearrangement as a resistance mechanism is extremely rare, although it has also been reported in this context [35]. 

## 4. Conclusions

Our case presentation underlines the importance of repeated testing for molecular alterations in the case of disease progression. The combination of afatinib and crizotinib in a patient with *EGFR*-, *HER*2-, *ROS1-* and *MET*-altered NSCLC led to a durable response and was well-tolerated after dose modification.

## Data Availability

All data generated or analysed during this study are included in this article. Further enquiries can be directed to the corresponding author.
